# Cumulative Risk Assessment in the Lorraine Region: A Framework to Characterize Environmental Health Inequalities

**DOI:** 10.3390/ijerph14030291

**Published:** 2017-03-10

**Authors:** Julien Caudeville, Despoina Ioannidou, Emmanuelle Boulvert, Roseline Bonnard

**Affiliations:** INERIS (French National Institute for Industrial Environment and Risks), 60550 Verneuil-en-Halatte, France; Desponia.IOANNIDOU-ETUDIANT@ineris.fr (D.I.); Emmanuelle.BOULVERT@ineris.fr (E.B.); Roseline.BONNARD@ineris.fr (R.B.)

**Keywords:** cumulative, exposure, spatial, environmental inequalities

## Abstract

The study explores spatial data processing methods and the associated impact on the characterization and quantification of a combined health risk indicator at a regional scale and at fine resolution. To illustrate the methodology of combining multiple publicly available data sources, we present a case study of the Lorraine region (France), where regional stakeholders were involved in the global procedures for data collection and organization. Different indicators are developed by combining technical approaches for assessing and characterizing human health exposure to chemical substances (in soil, air and water) and noise risk factors. The results permit identification of pollutant sources, determinants of exposure, and potential hotspot areas. A test of the model’s assumptions to changes in sub-indicator spatial distribution showed the impact of data transformation on identifying more impacted areas. Cumulative risk assessment permits the combination of quantitative and qualitative evaluation of health risks by including stakeholders in the decision process, helping to define a subjective conceptual analysis framework or assumptions when uncertainties or knowledge gaps operate.

## 1. Introduction

Humans are exposed daily to multiple chemical and non-chemical (e.g., biological, physical, or psychosocial) stressors. However, toxicological and epidemiological studies typically examine individual stressor-response relationships. Ideally, direct measures of exposure (e.g., biomarkers or personal monitoring data) would be available for all key stressors related to a common health effect throughout the critical time period of exposure and in the population of interest [1]. The exclusive use of biomarker data in cumulative exposure assessment efforts is currently not practicable when considering a large number of diverse chemicals due to analytical and resource limitations [2], especially when the assessment should cover a large territory. Environmental quality data are often available at a fine administrative or resolution level, and enable the building of environmental indicators on a regional scale. The definition of indicators for the identification and characterization of environmental inequalities depends on the reutilization of this type of data, which is very diverse by nature, with regard to its initial intended objectives. In France, this kind of data has already made it possible to highlight important regional disparities in the distribution of environmental quality [3,4]. To date, geographical information systems (GIS) technology has proven to be a powerful tool for dealing with various types of environmental data. Some studies integrate georeferenced measure monitoring or modeling data to estimate the exposure dose, and may include studies on various single environmental media, such as soil [5], water [6], and air [7,8], or a multimedia approach [9]. There will be cases where risk cannot be quantified in any meaningful or reliable way due to lack of representative data or missing source contributions. In order to reduce the spatial data representativeness problem (based on the lack of available data) and characterize associated uncertainty, more sophisticated methods of spatial analysis have been developed [10,11]. Qualitative approaches could also be used to overcome the complexity and data deficiencies that hinder quantitative approaches. Broad indicators using geographically based measures of exposure are used as an indicator of cumulative exposures from all of the potential chemicals associated with that site.

Cumulative risk assessment (CRA) is defined as a science policy tool for organizing and analyzing relevant scientific information to examine, characterize, and quantify the combined adverse effects on human health from exposure to a combination of environmental stressors [12]. The ultimate goal of cumulative risk assessment is to provide answers to decision-relevant questions based on organized scientific analysis, even if the answers, at least for the time being, are inexact and uncertain [13]. Cumulative risk assessment therefore involves the quantitative or qualitative evaluation of risks to health and/or the environment from multiple exposures, sources, and routes, while considering differential susceptibility or vulnerability of population subgroups [14]. Due to the limited availability of integrated data on multiple stressors, analytical complexity, and method limitations, exposure assessment is one of the main challenges for CRAs.

Assessing risk that includes multiple different risk factors is considerably more complex methodologically and computationally than aggregate risk assessments or single-effect cumulative risk assessments. The advantage of a decision index is the ease in converting highly multivariate technical information into a single number. The approach involves developing a composite score—or index—from measures of various risk dimensions [14]. Various environmental risk indexes have been developed and applied to ranking and comparative analyses [15,16,17]. Often, those indexes use surrogate measures for risk rather than actual calculations of the probability of adverse effects. There is relatively little experience in combining different types of risk. A key issue seems to be the need for method development in this area. Some approaches require synthesizing a risk estimate (or risk indication) by “adding up” risks from different parts of the risk dimension [18]. In these cases, risk assessment requires a common metric such as an exposure dose or hazard quotient. For example, emissions of both carcinogens and non-carcinogens are weighted by a toxicity factor, so they can be combined in a risk-based screening “score” for a particular geographic area by the Environmental Protection Agency’s (EPA) Office of Pollution Prevention and Toxics [18]. Finding a common metric for dissimilar risks is not a strictly analytic process, because judgments must be made as how to link two or more separate scales of risks. These judgments could involve a subjective conceptual analysis framework defined during a deliberative process, including stakeholders to make good decisions and generate operational actions adapted to the policy objectives. Unfortunately, methods used to combine indicators are sometimes selected in an arbitrary manner, with little particular attention paid to the data standardization procedure. This can lead to indices which overwhelm, confuse and mislead decision-makers and the general public.

The present study aims to explore spatial data processing methods and the associated impact on the characterization and quantification of a combined health risk indicator. To illustrate the methodology of combining multiple publicly available data sources, we present a case study of the Lorraine region (France), where regional stakeholders were involved in the global procedures for data collection and organization. We also explore technical approaches for assessing and characterizing human health risks associated with a subset of cumulative risk issues.

To achieve those objectives, combining a data process and transfer modeling with a spatial approach is fundamental, a prerequisite that implies the need to first overcome different scientific limitations:

- selecting and processing interest variables that could be built to associate and partly describe the source-effect chain;
- developing indicators that permit the combination of risk factors.

We describe here a CRA case study characterizing combined exposures to noise with chemical contaminations of water, air, and soil. The study’s aims are (1) to present an approach that utilizes existing data for comparisons across exposures and populations that could be useful for identifying at-risk populations; and (2) to explore the advantages and disadvantages of using data standardization methods.

## 2. Materials and Methods

### 2.1. General Approach

In France, environmental health inequalities are understood as the unequal geographic distribution of multiple exposure. No statewide data are available that provide direct information on exposures. Exposures generally involve transfer of chemicals from a source through the environment (air, water, soil, food) to an individual or population. For the purposes of the study, data relating to pollutant sources, releases, and environmental concentrations are used to build indicators of potential human exposures to pollutant. Pollution burden indicators should relate to issues that may be potentially actionable by stakeholders. Based on the regional context and data availability, four dimensions were identified and found consistent with criteria for exposure composite indicator development: water, air, soil, and noise. Then, four subindicators should provide a measure that is relevant to the dimension it represents in the context of the study objectives. The subindicators used should also represent widespread concerns related to pollution in Lorraine and provide a good representation of each component.

**Figure 1:**
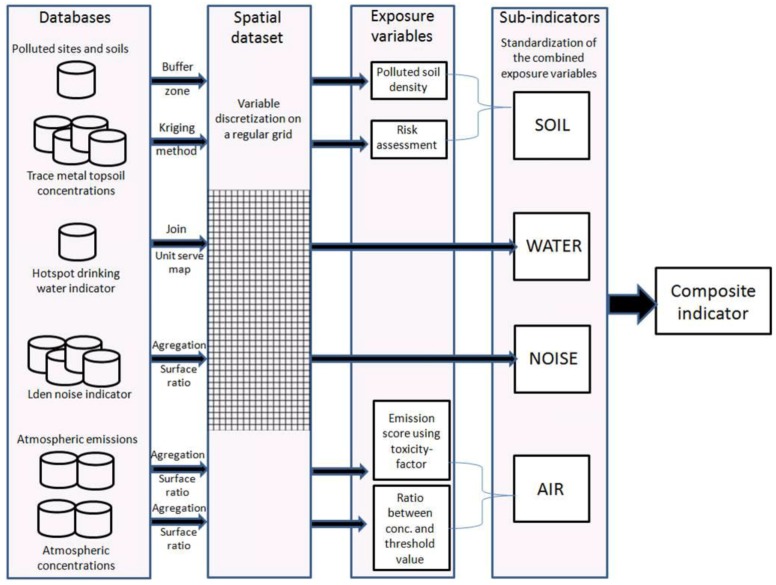
Conceptual framework of the data proceeding for determining composite indicators.

A composite indicator has to identify cumulative risk factor areas rather than hotspot areas of only one risk factor. In this cumulative risk assessment, the key aspect was to highlight areas where multiple stressors act together in contributing to risks. In this way, the strategy defined by the working group was to apply equity constraints for each risk factor. That means that each subindicator had to have a similar weight (equal average and range on the modeling domain) to build the composite indicator based on the sum of each standardized subindicators. The conceptual model is presented in Figure 1.

Aggregation of the different factor risks was made using different methodologies for discussing the impact of weighting and aggregation procedures on the effectiveness of risk maps for taking decisions for safeguarding citizen health. The subjective conceptual analysis framework was adopted using a deliberative process to define the common metric that would permit calculation of the composite indicator for dissimilar risks.

Data proceeding methods emerge from basic risk assessment concepts and is sufficiently expansive to incorporate multiple factors that reflect population impacts that have not been included in traditional risk assessments. A GIS-based modeling platform for quantifying human exposure (PLAINE: environmental inequalities analysis platform [9,19]) was used to build health risk indicators within the Lorraine region (France). The GIS-based platform permits researchers to:

- gather emission sources, environmental and population databases;
- discretize variables on a referent grid (data mapping);
- transform variables into exposure indicators (exposure transformation);
- derive subindicators by combining exposure indicators weighted by toxicological data or threshold values (data processing);
- build the composite indicator from standardized subindicators (indicator development).

### 2.2. Study Area

The case study concerns Lorraine, the north-eastern region of France, bordering Germany and comprising the departments of Meurthe-et-Moselle, Meuse, Moselle and Vosges. It is a moderately densely populated region, with an area of 23,547 km^2^ and 2.5 million inhabitants—4% of France’s total population—making it the 11th most populous region in the country. It ranks eighth in GDP (gross domestic product) among the 26 regions of France, placing it per capita among the top economic producing regions in the country, along with Alsace and Île-de-France (Paris). From the end of the 19th century to the 1960s, the economic development of Lorraine was built on two dominant industries: coal mining and steel production. The logistics and service sectors have experienced the strongest growth in recent years, while traditional industries have undergone a decline. Consequently, the region has experienced major difficulty with increasing unemployment, although it is still below the national average. In 1997 the last iron ore mine in Lorraine, which once produced over 50 million tons of iron, was closed.

### 2.3. Stakeholder Involvement

Each region drew up a Regional Environment and Health Action Plan to implement the main objectives of the French National Action Plan according to its own specific needs. Different regions in France, including Lorraine, have included environmental health inequalities reduction in their planning, and need assessment to guide priorities for voluntary action.

Transparency of decision making and policy development is the cornerstone of environmental inequalities reduction action. In that spirit, a working group consisting of regional representatives of environmental database managers, thematic experts, and environment and health regional stakeholders (see acknowledgements) was created to define the study objectives definition and the conceptual framework. This group was particularly involved in data collection, selecting stressors, and ensuring the adequacy of the assessment results with potential action implementations.

### 2.4. Data Selection

A wide range of data on different sources, agents, pathways and media might potentially be required for integrated assessment of environmental health risks. These data might be used as inputs for models or proxies for other exposure metrics. The decision was made to focus on sources relevant either directly (as measures of exposure) or indirectly (as potential input variables for modeling). In addition, population data were included, since this provides important proxies for source activity in many instances and is, of course, an essential component of exposure assessment.

A data inventory was made reflecting the following main themes: soil, land cover, air, drinking water quality, atmospheric emissions/concentrations, polluted sites, and soil and exposure factors. In order to make the task manageable, attention was initially focused on data available at the regional scale that are gathered on a routine basis. Nevertheless, geographic coverage or extent, for example, is inevitably ambiguous. Indeed, most environmental data are samples and do not provide complete area coverage. Hence, in many cases, approaching full regional coverage is possible if different datasets are combined. Some data for Lorraine is a subset of the national monitoring network, so the density of sampling across Lorraine may be sparse. In this study, data sources have generally been included when they were considered to represent a potential basis for assessing exposures across populations at the regional level, either directly or by interpolation. From this inventory, the selection of the database was made based on the interests of and uses for this study (Table 1).

## 3. Data Processing

### 3.1. Data Spatialization

A share-of-population census, monitoring, and modeling of environmental quality data production were conducted independently of each other in accordance with specific needs and constraints. This discrepancy implies that the different data types from different sources and support databases cannot be directly represented under a common denominator, namely their spatial location or distribution. Representation is therefore achieved by depicting the different data types as layers and superposing those layers in the same geographical reference grid. The problem of linking data sets derived from incompatible spatial frameworks (for example, linking point- and pixel-based environmental data) has attracted considerable attention. A referent grid of 1 × 1 km was generated for the study, and all the spatial variables were discretized on this grid. Tools have been developed using modeling, spatial analysis, and geostatistic methods to build and discretize interest variables from different supports and resolutions on the 1 km^2^ regular grid within the Lorraine region. For example, surface soil concentrations were estimated by developing a kriging method able to integrate surface and point spatial supports [11]. For water, distribution unit serve maps were used to spatialize water data measured at water treatment plants. Modeled or estimated noise and air variables were aggregated from their initial grids to the referent grid using surface ratios. Buffer zones around potentially contaminated sites and soils were generated using a distance (300 m) defined by the working group. GIS was used to partition the proximity data assigned to the areal unit of the referent grid that is only partially within the distance buffer into “inside the buffer” and “outside the buffer” portions based on the percentage of the areal unit that lies within and without the distance buffer, respectively.

### 3.2. Exposure Transformation

Different methods were used to transform environmental spatial datasets into exposure variables. An exposure model developed by INERIS (MODUL’ERS [22]) was used to assess the transfer from soil to individual exposure through ingestion pathways (soil and vegetation pathways). This model was used to estimate population age class hazard quotients (HQ) from interpolated topsoil trace metal concentrations and for estimating non-cancer risk. For the ingestion pathway, the HQ is the ratio of the average daily dose (ADD; milligrams per kilogram per day) of a chemical to the reference dose (RfD, milligrams per kilogram per day), defined as the maximum tolerable daily intake of a specific pollutant that does not result in any deleterious health effects.

Generally, to combine HQs, stressors need to have a common target organ [14]. We assumed independence of action and we summed the HQs to build the topsoil concentration indicator. Use of this exposure model to map exposure indicators can be seen in detail in Caudeville et al. [9]. The air concentration indicator was estimated using the sum of the ratio between the annual average pollutant atmospheric concentrations and the European air quality standards [23]. Broad indicators were built using geographically based measures of hazard as a cumulative measure. For example, we used distance from a polluted soil site to build a proxy based on the density of the potentially contaminated site by areal unit. A score was used to estimate the relative risks of direct emissions by combining total pollutant emissions (sum of pollutants) and toxicity-weighted pollutant emissions for cancer or respiratory non-cancer effects. Weighting emissions by toxicity does not take into account fate, transport, or location and behavior of receptor populations. It is often desirable to aggregate indicators into broader thematic indices. The air risk factor indicator combined modeled concentrations and estimated emissions following the equity principle to give similar weights to the two dimensions with similar area numbers, global indicator averages and indicator ranges. Site proximity and topsoil concentration databases were also combined into a higher-level soil indicator simply by adding them.

For water, drinking water concentrations were compared to European drinking water standards (chosen previously by a different working group) in a tool developed by the Regional Health Agency of Lorraine. The four-year averaged number of substance exceedance thresholds permitted us to build a score. An elevated score indicates that drinking water supplied in those areas could have concentrations that could lead to chronic disease in the population. The link between exposure and outcome (other terms: endpoint, reaction, response) was given by reasonably well-established exposure-response curves which are derived from research into noise effects. The Lden indicator (developed in the context of the noise European framework) was used to map noise around road infrastructures. It corresponds to the average sound pressure level over all days, evenings and nights in a year.

### 3.3. Data Transformation and Indicator Development

The standardization procedure described here subjects subindicators to two different transformations that yield dimensionless and comparable figures. These can readily be aggregated to a higher-level thematic indicator simply by adding them. Aggregation of the different risk factors was made using different methodologies to discuss the impact of weighting and aggregation procedures on the effectiveness of risk maps used for making decisions safeguarding citizen health. Two methods were explored to build a homogeneous metric that permitted us to respect the equity constraint defined by the working group.

The first method used a normal score function applied to transform each dataset into a normal distribution varying between 0 and 1. A score was assigned for each geographic unit derived from the ranks of the observations within the dataset. For each individual grid, a value was assigned which either expressed exactly or approximated the order statistic expectation of the same rank in a sample of standard normal random variables with the same size as the observed data set. The second method assigned a percentile, varying between 0 and 1, for each subindicator and geographic unit, based on the rank order of the value. A percentile was calculated from the ordered values for all areas that have a score.

When a geographic area had no indicator value (for example, an area that had no noise estimation) or had exposure values equal to zero (for example, an area with no water exposure hotspot), a background exposure value was assigned corresponding to the mean of the first missing transformed ranks. This approach permitted us to obtain data independent of the chosen unit and scale with a similar average and range for each subindicator. Those scores allow comparison of one geographic area to other localities in the region where hazard effect data or population characteristics are present. Thus each area’s score for a specific indicator is relative to the ranks of that indicator in the rest of the region.

The mathematical formula for calculating the composite indicator of the two methods used addition of the normalized or percentile-ranked subindicators. The method used existing environmental data to create a screening score for the population across the area. The population size at fine resolution was used to weight the composite indicator spatial aggregation at the French census block level. An area with a high score would be expected to experience much higher impacts than areas with low scores.

## 4. Results

One distinctive aspect of CRAs is their ability to examine multiple stressors that may affect health outcomes. Excluding non-chemical stressors from analysis may underestimate cumulative exposure and/or risk [1]. We illustrate a method that utilizes publicly available data sources and existing analytical methods to examine chemical and non-chemical stressor exposures to inform screening-level CRAs in order to identify subpopulations that may have a higher level of concern. Our method uses a combination of toxicological/threshold values and data transformation methods to characterize the unequal geographic distribution of environmental risks.

### 4.1. Indicator Mapping

**Figure 2:**
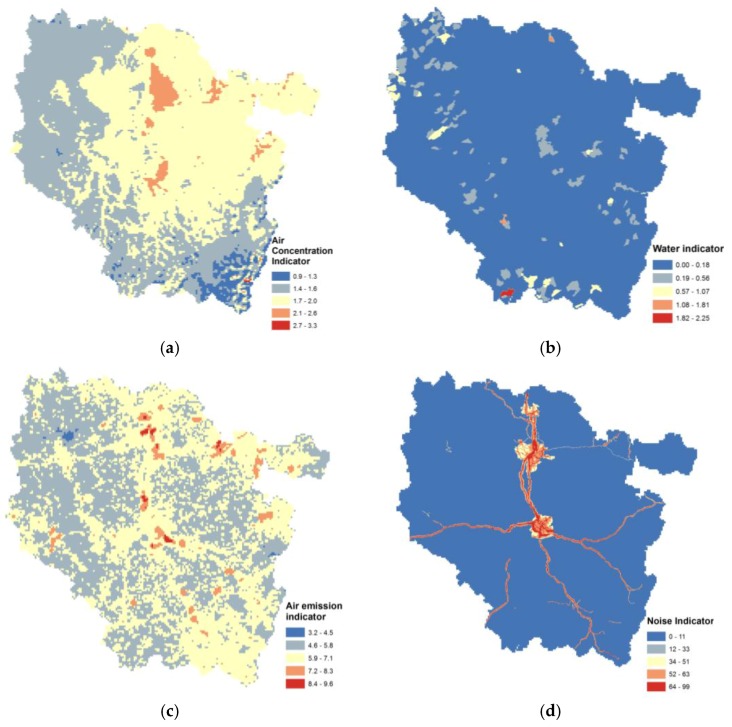

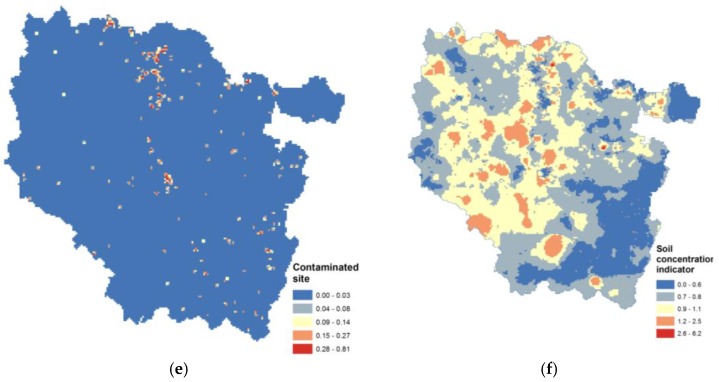
Maps of exposure variables for: (**a**) air concentration; (**b**) water exposure hotspots; (**c**) air emissions; (**d**) LDen noise; (**e**) potential site and soil contamination; and (**f**) soil concentration.

Subindicators are presented here as regional maps (Figure 2).

The air concentration indicator variations are weak throughout the studied area due to the background exposure concentration (Figure 2a). The area with the most elevated values corresponds to urban agglomerations. For drinking water, the map presents several hotpots corresponding to one or a combination of different pollutant concentrations above defined thresholds (Figure 2b). For example, the most elevated area of concentration (2.25) corresponds to natural arsenic and fluoride exceedances averaged during the four-year period of the study. The atmospheric emission indicator (Figure 2c) presents a similar pattern to that of the air concentration, but it also integrates district-level data in the higher-value district where an industrial site is located. The most elevated value (9.5) corresponds to polycyclic aromatic hydrocarbon and benzene emissions from steel industry activity. The noise map (Figure 2d) presents no value (as 0) for 90% of the studied area. Existent values are located on the region’s principal roads, based on available modeled noise levels. Principal contaminated sites and soil are located around a north-south axis called the Lorrain furrow (Figure 2e). The soil concentration indicator map presents two areas in which the value is greater than four (Figure 2f). These correspond to well-known contaminated sites. The largest value corresponds to a topsoil contamination of Hg, Cd, Cu and Zn, and the other to elevated concentrations of Cd in the topsoil. Specific spatial patterns are influenced by data spatialization methods and exposure variable transformation. Spatial resolution could also have an impact on individual area indicator values. In contrast to water risk factor indicators, where spatialization corresponds to a surface ratio of the initial spatial layer, the distribution of the emission indicators depends on the size of the geographic support aggregation.

**Figure 3:**
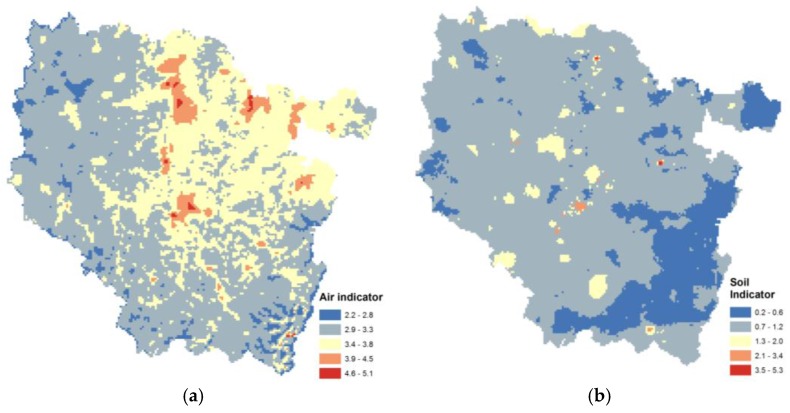
Maps of combined exposure variables for (**a**) air and (**b**) soil.

Maps of the combined exposure variable indicators for air and soil are presented in Figure 3. Exposure variables of contaminated sites and soil and topsoil concentration data were combined in order to integrate the soil contribution of rural areas (in this database, topsoil concentration samples are mainly located in forests and agricultural fields) and urban areas (contaminated sites and soil are historically located in urban areas). The atmospheric emissions and concentration dataset were combined in order to take into account three conventionally estimated pollutant concentrations (O_3_, PM_10_, NO_2_) and the emissions of 24 pollutants, for better taking into account industrial sources.

**Figure 4:**
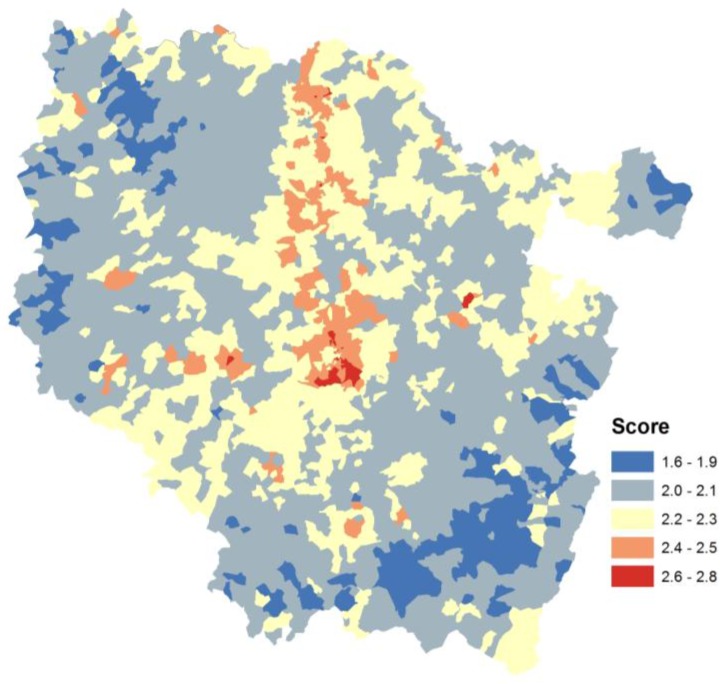
Composite indicator map (SN method) aggregated at the French census block level.

The largest risk expressed by the composite risk indicator, obtained using the normal transformation method, corresponds to an industrial site. Spatial patterns of hotspot exposure are localized on the Lorrain furrow, reflecting the association between regional industrial and organized urban space dynamics (Figure 4).

### 4.2. Data Processing and Indicator Development

The composite indicator of spatial patterns depends on the local combination of the individual subindicators and their local interrelationships. Table 2 presents the correlation coefficients (*r*) obtained between the different estimated risk factor indicators.

Weak correlations were found between the subindicators. The highest correlation (*r* = 0.365) corresponds to noise and air, due to similar environmental sources (automobile transport). The regression analysis revealed low negative correlations between water and the other subindicators. This subindicator is therefore less implicated in the highest composite indicator values.

**Figure 5:**
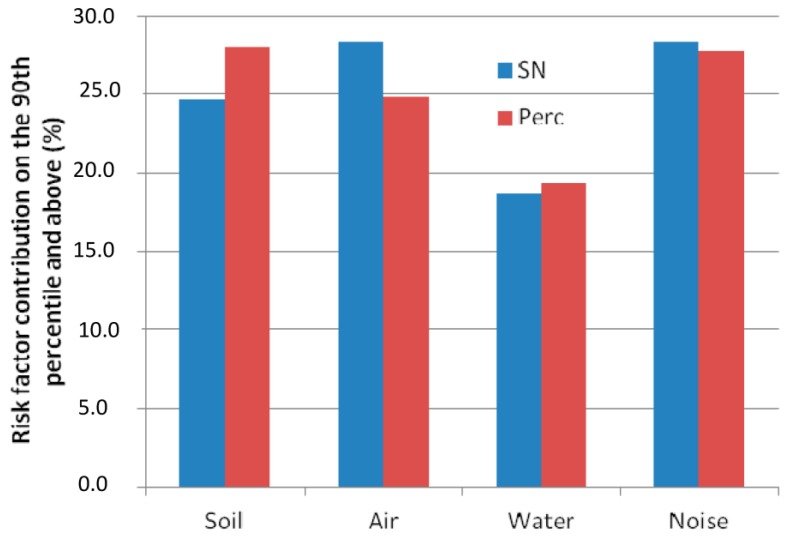
Histograms of the percentage contribution of the risk factor to the composite indicator above the 90th percentile for the normal transformation (SN = blue) and percentile rank method (Perc = red).

**Figure A1:**
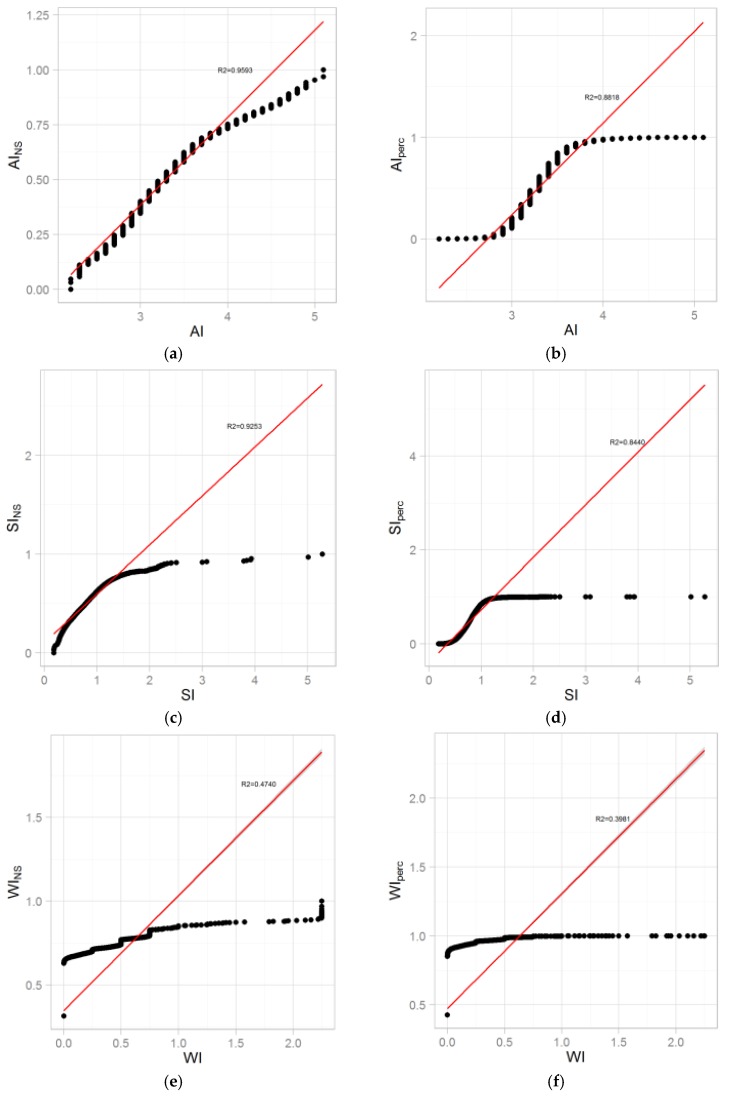

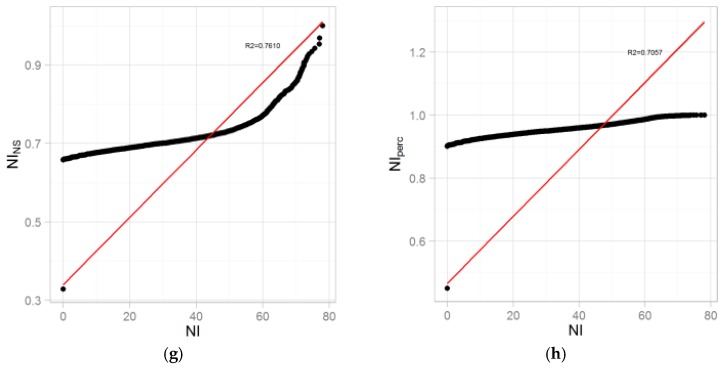
Correlation between subindicator before and after data transformation: (**a**) air and normal transformation; (**b**) air and percentile rank transformation; (**c**) soil and normal transformation; (**d**) soil and percentile rank transformation; (**e**) water and normal transformation; (**f**) water and percentile rank transformation; (**g**) noise and normal transformation; (**h**) noise and percentile rank transformation.

Figure 5 shows subindicator contributions above the 90th percentile for the composite indicators estimated using the two transformation methods. The noise and water risk factor contributions are quite similar. The variation of soil and air risk factors may be explained by the slope curve impact on the resulting composite indicator. Air and soil curves have similar forms for the percentile rank method (see Appendix A, Figure A1b,d), in contrast to the normal transformation for which the slope on the maxima range is more flattened for the soil than for the air risk factor (see Appendix A, Figure A1a,c). This results in a stronger contribution of the air risk factor in the composite indicator based on normal transformation compared to uniform transformation.

## 5. Discussion

Efforts were made to select complete, accurate and current datasets for inclusion. Nonetheless, there are different kinds of uncertainty that are likely to be introduced in the development of this type of approach. Those uncertainties mainly depend on: (1) the combination method’s impact on the capacity of the selected indicator metric to reflect the considered phenomena; (2) data representativeness, which controls the degree to which data gaps or omissions influence the results. The latter mainly concerns missing spatio-temporal aspects, source contributions, and the characterization level of the exposure scheme (from the source to the external exposure modeling with the integration or not of transfer/transport phenomena and population behavior).

Empirical methods could be set up, driven by the will to characterize other contributions not integrated in an initial database. Those choices are guided to reach the best compromise between data representativeness and method robustness, consistent with the objectives of the study.

In chemical mixtures risk assessment, exposure addition, more commonly called dose addition, assumes a common toxic mode of action across compounds, or at least evidence of toxicologically similar responses, so that the “total dose” is of concern for the assessment. Where only qualitative data is available, proxy indicators can be built, but are more difficult to use for measuring exposure quantitatively and for combining with exposure assessment variables. In our study different options were proposed and adopted by the working group.

Two data transformation methods were applied to provide a common metric for each subindicator (a single function applied to each *X* or each *Y* data value) with respect to the equity constraint. The indicators used in this analysis have varying underlying distributions, and distribution normalization or percentile rank calculations provide a useful way to transform data. Nevertheless, the choice of a transformation implies the making of assumptions about those distributions (normal for the normalization or uniform for the percentile rank transformation method) that control the degree to which the data that are included in the model are correct.

Therefore, each area’s value for a specific indicator is relative to the distance to the average in the data space in the rest of the places in the region. The distribution form used will impact the weight of an individual area in the resulting composite indicator. In our case, where cumulative hotspot exposure is the desired measure, better characterization of the highest values is researched.

The transformation needed to reproduce the relative distance between each point of the original subset reflects the efficiency of this function to limit the over- or underestimation of a range of points. As the sigmoid form shows (see Appendix A, Figure A1b), rank percentile transformation smooths the extremum values. Because the composite indicator objective is used to highlight potential hotspot exposure, the highest value distance respects are the most important/critical. In the test case, the normal function permits a better description of the outliers. An over- or underestimation will impact the global ranking of other individual areas or subindicator weightings. The adequacy of the expressed relative distance between points on a specific curve range could be characterized by the curve slope (a low slope implies a potential underestimation).

Our environmental noise estimates were only based on modeled noise levels from road traffic. Since industries, railroads and an airport also exist within the study area, it is likely that road traffic is not the only main contributor to human activity–related environmental noise in this region. While environmental noise may be the primary source of background noise in communities, non-environmental sources of noise may also be present and influence individual-level noise estimates. In this study, background exposure was not taken into account due to data deficiency. For water-related data, background exposure was also not integrated, due to the subindicator calculation mode where water concentrations below substance-specific thresholds were not considered.

Ranking the data involved putting the values in numerical order and then assigning new values to denote where in the ordered set they fall. In those two datasets there are ties in the data where no value or no hotspot exposure is considered, expressed as a zero value. This means that several values are the same, so that there is no strictly increasing order. For the considered background exposure, we averaged the ranks for the tied values (see Appendix A, Figure A1e–h). This processing resulted in a heteroscedasticity creation (unequal variances) that impacted the local contributions of associated subindicators on the resulting composite indicator. Affecting a background exposure value will generate a gap between the initial null value and the rest of the distribution. It tends to flatten the curve slope, reduce the distance between points in the maxima value range, and finally decrease subindicator sensibility in the resulting composite indicator.

In order to choose a calculation design in the context of environmental inequalities, certain queries need to be answered. One of these is to provide a uniform basis for mapping that is fine enough to reflect local variations in exposures, both to aid visual representation and interpretation of the data and to facilitate analysis of spatial patterns. Regular grid systems generally best satisfy that criterion and permit us to reduce the so-called “small number problem”, which can lead to highly unstable estimates of risk and large variations in uncertainty between zones [24].

More specifically, we need to define a calculation mode in an attempt to overcome scientific knowledge gaps in combining quantitative and qualitative approaches. A subjective conceptual analysis framework was set up during a deliberative process including stakeholders. This included the need to traduce the working group’s adopted “rules” in terms of calculation assumptions and designs. For example, the equity constraint proposed here requires for each subindicator a common metric with a similar mean and range. Different distributions are permitted with respect to this constraint, such as family symmetrical distributions (uniform, normal, logistic, etc.), which can be used during the standardization procedure. The selection of the distribution must be led, in order to reduce the distance between point distortions generated during the standardization procedure as much as possible. This can be achieved by estimating the best fitting distribution by ranking goodness-of-fit statistics using Anderson–Darling or Kolmogorov–Smirnov tests. If better precision is desired at a specific range of the distribution (as the extremum values), a sliding window regression could be used to compute slope estimates along with the curve and help interpret the potential impact of the transformation on the individual subindicator area value. The distribution selection process must also take into account the impact of heteroscedasticity that arises by assigning default values for background exposure or areas where values are missing. This might be measured by comparing the ratio between the assigned and the first unassigned default values. Higher variance differences will decrease the sensibility of the subindicator in the composite indicator. In our test case, normal transformation was preferred over uniform standardization. However, the choice of calculation design is sometimes a compromise between potential competing needs.

## 6. Conclusions

This pilot study successfully applied a composite risk indicator using a cumulative screening method at a fine resolution in Lorraine. The issues confronted when considering such a wide range of different data sources provided insight into ways to improve data generation and collection. However, we encountered several limitations in regards to specific indicators. Indicators are surrogates for the characteristics being modeled, so a certain amount of uncertainty is inevitable. That means this model, comprised of a suite of indicators, is considered useful in identifying places burdened by multiple sources of pollution. Qualitative approaches may be used to overcome the complexity and data deficiencies that hinder quantitative approaches. Cumulative risk assessment permits the combination of quantitative and qualitative evaluation of health risks by integrating stakeholders in the decision process of defining the subjective conceptual analysis framework or assumptions when uncertainty or knowledge gaps operate. Engaging stakeholders associated with the development, review, and use of exposure-science information contributes to formulating problems, collecting data, accessing data, and developing decision-making tools.

Using a limited data set, a test of the model’s assumptions to changes in subindicator spatial distribution showed the impact of data transformation in identifying more impacted areas.

Our results permitted us to identify pollutant sources, determinants of exposure, and potential hotspot areas. A diagnostic tool was developed for stakeholders to visualize and analyze the composite indicators in an operational and accurate manner. The designed support system will be used in many applications and contexts:

- mapping environmental disparities throughout the Lorraine region;
- identifying vulnerable populations and determinants of exposure, to set priorities and target for pollution prevention, regulation, and remediation;
- providing exposure databases to quantify spatial relationships between environmental, socioeconomic and health indicators.

Over the next few years, we plan to refine the method by using spatial models to combine the global source–exposure–effect chain and to integrate additional indicators more adapted for agricultural or urban contexts (such as pesticide substances or radiofrequency exposure). In addition, we will look for new ways to integrate population mobility into exposure estimations. Exposure indicators and data processing algorithms will be integrated in the French coordinated integrated environment and health platform PLAINE to map and analyze environmental health inequalities at the national scale.

## Figures and Tables

**Table 1:** Data sources included in the study.

Dimension	Specific Aspect	Data Source
Noise	LDEN noise indicator, dB(A)	DDT(M) (French Departmental Directorates for Territorial (and Sea) Administration) and the three biggest agglomerations (Metz, Nancy et Thionville) of the Lorraine region (200 m, 250 m, 1 km grid, annual average LDen indicator calculated from transport sources, 2012)
Soil	Contaminated sites and soil	MEDDE (French Ministry of Ecology, Sustainable Development and Energy): listing of sites requiring preventative or curative action by the administration. 322 sites were integrated for the Lorraine region in 2013
	Nickel, Cadmium, Chromium, Lead, Arsenic, Mercury, Copper topsoil concentrations	French Chamber of Agriculture, INRA (French National Institute of Agronomic Research), BRGM (French Bureau of Geological and Mining Research). Topsoil trace metal topsoil concentration databases (BD ETM-Trace Metal database and RMQS-Soil Quality Monitoring Network) [20]. Around 8000 localizations for each pollutant from 1995 to 2013
Water	Indicator of exceeding thresholds for 500 measured parameters in the drinking water	ARS (Health Regional Agency of Lorraine): Indicator based on the Black point–Grey point to highlight the area where pollutant concentrations are elevated, from 2007 to 2011. Geocoding using the Sise’eaux database, the administrative boundary map of France and distribution unit serve map [21]
Air	NOx and Particulate Matter (PM_10_) atmospheric concentrations, number of daily exceeding threshold by year for ozone	Official Air Quality Monitoring Association of Lorraine (AASQA). Annual average concentration modeled from the regional register of atmospheric pollutant emissions for PM_10_ and NOx, number of daily exceeding threshold by year for ozone within the Lorraine region (1 km grids) and in focus areas (100 m), 2011
	24 pollutants atmospheric emissions	Official Air Quality Monitoring Association of Lorraine (AASQA). Regional register of pollutant atmospheric emissions (1 km grid and district administrative level, 2006)
Population size	Population size at 0.04 km^2^ grid	French National Institute of Statistics and Economic Studies (INSEE), 2008

**Table 2:** Spearman correlation coefficients (*r*) between the different risk factor indicators estimated.

Risk Factor	Noise	Water	Soil	Air
Noise		−0.047	0.017	0.365
Water	−0.047		−0.01	−0.114
Soil	0.017	−0.01		−0.005
Air	0.365	−0.114	−0.005	

## Appendix A

Linear regressions on the original data were used to test the assumption efficiency of the underlying transformation (Figure A1). For air and soil, the normal transformation (Figure A1a,c, *R*^2^ = 0.96 and 0.93) presents a better representation than the rank percentile transformation (Figure A1b,d, *R*^2^ = 0.88 and 0.84). This is due to the original distribution form of the subset, which better fits a normal distribution than a uniform distribution.
